# Carotid geometry is independently associated with complicated carotid artery plaques

**DOI:** 10.3389/fcvm.2023.1177998

**Published:** 2023-06-12

**Authors:** Christoph Strecker, Anna Kopczak, Tobias Saam, Dominik Sepp, Anja Hennemuth, Ernst Mayerhofer, Sven Poli, Ulf Ziemann, Holger Poppert, Andreas Schindler, Andreas Harloff

**Affiliations:** ^1^ Department of Neurology and Neurophysiology, Medical Center—University of Freiburg, Faculty of Medicine, University of Freiburg, Freiburg, Germany; ^2^ Institute for Stroke and Dementia Research, University Hospital, LMU Munich, Munich, Germany; ^3^ Die Radiologie, Rosenheim, Germany; ^4^ Department of Neuroradiology, Klinikum rechts der Isar, Technical University of Munich (TUM), Munich, Germany; ^5^ Institute for Imaging Science and Computational Modelling in Cardiovascular Medicine, Charité-Universitätsmedizin Berlin, Berlin, Germany; ^6^ Department of Neurology, Massachusetts General Hospital, Boston, MA, United States; ^7^ Department of Neurology and Stroke and Hertie Institute for Clinical Brain Research, University of Tübingen, Tübingen, Germany; ^8^ Department of Neurology, Helios Klinikum München West, Munich, Germany; ^9^ Institute of Neuroradiology, University Hospital, LMU Munich, Munich, Germany

**Keywords:** carotid artery, atherosclerosis, carotid geometry, complicated plaque, magnetic resonance imaging, multicontrast plaque imaging

## Abstract

**Introduction:**

Complicated carotid artery plaques (cCAPs) are associated with an increased risk of rupture and subsequent stroke. The geometry of the carotid bifurcation determines the distribution of local hemodynamics and could thus contribute to the development and composition of these plaques. Therefore, we studied the role of carotid bifurcation geometry in the presence of cCAPs.

**Methods:**

We investigated the association of individual vessel geometry with carotid artery plaque types in the Carotid Plaque Imaging in Acute Stroke (CAPIAS) study. After excluding arteries without plaque or with insufficient MRI quality, 354 carotid arteries from 182 patients were analyzed. Individual parameters of carotid geometry [i.e., internal carotid artery (ICA)/common carotid artery (CCA) ratio, bifurcation angle, and tortuosity) were derived from time-of-flight MR images. The lesion types of carotid artery plaques were determined according to the American Heart Association classification of lesions by multi-contrast 3T-MRI. The association between carotid geometry and a cCAP was studied using logistic regression after adjusting for age, sex, wall area, and cardiovascular risk factors.

**Results:**

Low ICA/CCA ratios (OR per SD increase 0.60 [95%CI: 0.42–0.85]; *p* = 0.004) and low bifurcation angles (OR 0.61 [95%CI: 0.42–0.90]; *p* = 0.012) were significantly associated with the presence of cCAPs after adjusting for age, sex, cardiovascular risk factors, and wall area. Tortuosity had no significant association with cCAPs. Only ICA/CCA ratio remained significant in a model containing all three geometric parameters (OR per SD increase 0.65 [95%CI: 0.45–0.94]; *p* = 0.023).

**Conclusions:**

A steep tapering of the ICA relative to the CCA and, to a lesser extent, a low angle of the carotid bifurcation were associated with the presence of cCAPs. Our findings highlight the contribution of bifurcation geometry to plaque vulnerability. Thus, assessment of carotid geometry could be helpful in identifying patients at risk of cCAPs.

## Introduction

Complicated carotid artery plaques, i.e., American Heart Association lesion type (AHA-LT) VI carotid artery plaques (1–3), are associated with an increased risk of future cerebrovascular events as recently demonstrated in the Carotid Plaque Imaging in Acute Stroke (CAPIAS) study (4). Furthermore, such atheromas were three times more frequent ipsilateral to the side of the symptomatic compared with the asymptomatic hemisphere in patients with acute cryptogenic stroke, underlining the embolic potential of these atheromas (5). Since vascular risk factors affect both carotid arteries similarly, there must be other factors that determine unilateral atheroprogression toward a complicated plaque.

For the optimal prevention of ischemic stroke, it is important to understand why a stable plaque turns into a complicated plaque independent of cardiovascular risk factors and degree of stenosis. Recent studies (6–9) demonstrated that a wide bulb of the ICA, a large carotid bifurcation angle, and low wall shear stress (WSS) are independent predictors of wall thickening in early atherosclerosis. High WSS is associated with plaque instability and rupture by endothelial denudation, fibrous cap thinning, intraplaque hemorrhage, and thrombus formation, thereby turning a stable plaque into a complicated plaque (10, 11). Consistently, Tuenter et al. (12) demonstrated an independent association of intraplaque hemorrhage, the most frequent feature of complicated plaques (2), with high WSS using computational fluid dynamics (CFD) in 79 patients with asymptomatic carotid plaques. Similarly, Zhang et al. showed that WSS was significantly higher in regions adjacent to high-risk than low-risk plaques using 4D flow MRI (13). Moreover, Sadat et al. (14) studied peak stresses within the plaques using *in vivo* MRI in combination with finite-element analysis in each of the 25 patients with acute or recent stroke and carotid stenosis. They asserted that maximum stress was significantly higher in patients with acute symptoms than that in patients with symptoms weeks ago, even in a lower degree of internal carotid artery (ICA) stenosis, indicating that mechanical forces of blood flow can form complicated plaques.

Measuring carotid WSS or plaque stress could be useful to identify patients at risk of plaque rupture prior to the stroke. However, these methods are not suited for clinical routine due to the time-consuming techniques such as 4D flow MRI or CFD requiring complex data analyses. By contrast, vascular geometry is an “easier-to-measure” surrogate of such hemodynamics because geometry shapes the flow (15). Thus, recent studies proposed that carotid geometry assessed by CT or MR angiography may serve as a surrogate parameter for critical flow in early (6, 8, 16) and advanced (17, 18) stages of atherosclerosis. Accordingly, Jiang et al. (17) demonstrated an independent association of carotid geometry, i.e., a smaller lumen expansion at the carotid bifurcation, with vulnerable plaques in 501 carotid arteries of 501 patients in the cross-sectional Chinese Atherosclerosis Risk Evaluation II (CARE II) study.

However, the study of Jiang et al. (17) did not include carotid arteries with luminal stenosis, and a similar study on Caucasians, who show differences in the distribution of carotid atherosclerosis and the incidence of complicated carotid artery plaques (19) compared to Asians, has not yet been performed. Thus, we investigated the interrelation of ICA/common carotid artery (CCA) ratio, bifurcation angle, and tortuosity with the incidence of complicated carotid artery plaques (cCAPs) based on MRI data in a larger multicenter study of Caucasians with acute ischemic stroke.

## Methods

### Study population

We analyzed a subset of the Carotid Plaque Imaging in Acute Stroke (CAPIAS) study (ClinicalTrials.gov: NCT01284933) that prospectively recruited patients with acute ischemic stroke in the territory of one carotid artery and unilateral or bilateral carotid artery plaques with a plaque thickness of at least 2 mm at any side. Patients with carotid artery stenosis of ≥70%, according to NASCET criteria, were excluded. CAPIAS was conducted at four large tertiary stroke centers in Germany: Ludwig Maximilian University Munich, Technical University of Munich, Eberhard Karl University of Tuebingen, and Albert Ludwig University of Freiburg. The study design and inclusion criteria have been published previously (5, 20). The study was approved by the responsible ethics committees, and all participants provided written informed consent.

In our present analysis, we included only carotid arteries with plaques and MRI data with sufficient image quality to assess bifurcation geometry.

### Carotid plaque imaging

All participants were examined with a 3-Tesla MRI scanner (Magnetom Verio, Skyra, Tim Trio, Prisma or Biograph mMR, Siemens Healthineers, Erlangen, Germany) and a four-channel surface coil (Machnet, Eelde, Netherlands). We obtained time-of-flight (TOF) MR angiographs and axial pre- and postcontrast black-blood T1-, PD-, and T2-weighted sequences with fat suppression and an in-plane resolution of 0.5 mm^2^ as described previously (5, 20, 21). Carotid plaque composition was classified according to the modified AHA-LT criteria (22) by two experienced radiologists (TS and AS) blinded to the patients' characteristics (5). Plaques were defined as AHA-LT III if they showed a small eccentric plaque without calcification or a diffuse intimal thickening; AHA-LT IV/V plaques were characterized by a lipid-rich necrotic core surrounded by fibrous tissue with possible calcifications; complicated AHA-LT VI plaques were defined by the presence of a surface defect, intraplaque hemorrhage, or thrombus; and AHA-LT VII plaques were calcified plaques. There were no AHA-LT VIII plaques without lipid core and with possible small calcifications in our study sample (22). Wall area measurements were performed with a custom-designed semiautomatic image analysis tool (CASCADE, University of Washington, Seattle, Washington) (23).

### Geometry analysis

**Figure 1:**
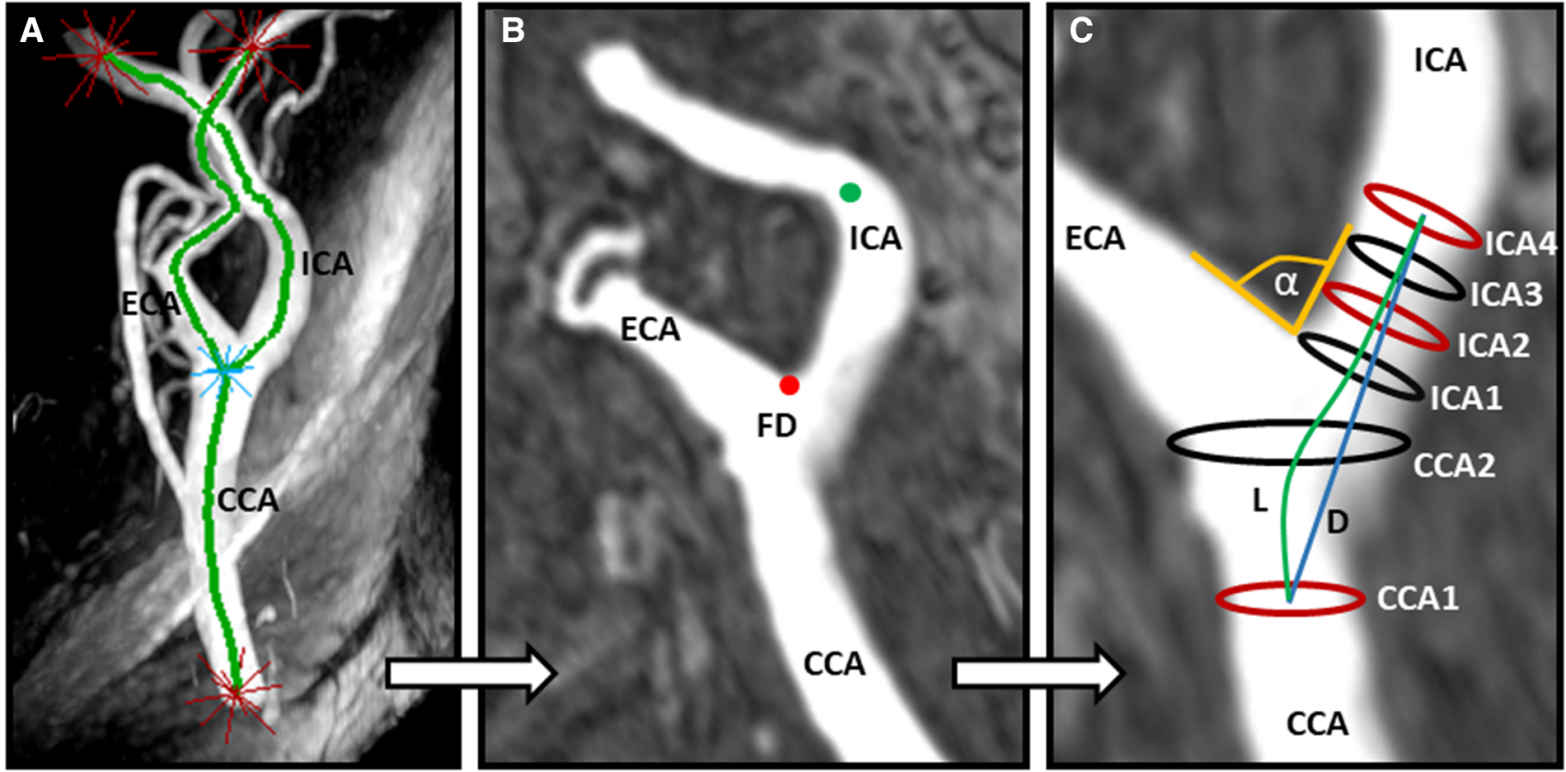
Assessment of carotid artery geometry. (A) Computation of a centerline based on time-of-flight (TOF) MR angiography after manually determining starting and end points (labeled as red stars). (B) Manual labeling of the ICA (green dot) and identification of the flow diverter (FD, red dot). (C) Automatic computation of three geometry parameters: *α* indicates the bifurcation angle; ICA2 and CCA1 represent the maximum diameters for calculation of the ICA/CCA ratio; L and D indicate the CCA–ICA distances along the lumen center (**L**) and direct connection (**D**), respectively, for the calculation of carotid tortuosity. CCA, common carotid artery; ICA, internal carotid artery; ECA, external carotid artery.

TOF data sets were imported into CaroTo, an extension of the MEVISFlow research software (Fraunhofer MEVIS, Bremen, Germany) (24). A centerline was created in each carotid bifurcation after the manual definition of CCA, ICA, and external carotid artery (ECA) as start- and endpoints for centerline computation. Consecutively landmarks including the flow diverter (FD) and ICA were marked (Figures 1A,B). From these landmarks, predefined points were automatically generated on the centerline, indicating the level of each analysis plane. Analysis planes were manually corrected to align perpendicular to the carotid lumen if necessary. This resulted in six cross-sectional planes (Figure 1C): plane 1 on the CCA centerline 1 cm below the flow diverter (CCA1); plane 2 within the carotid bulb between CCA1 and the flow diverter (CCA2); and planes 3–6 along the ICA with the starting point at the FD and oriented perpendicularly to the centerline with a spacing of 3 mm each (ICA1–ICA4). Geometric parameters have been previously described and successfully applied (7, 8, 25) and included (a) ICA/CCA diameter ratio; (b) bifurcation angle (measured by two tangential lines of the first 1 cm of the outer wall of the ECA and ICA starting at the FD); and (c) CCA-ICA tortuosity (ratio of the centerline connection and the direct line of plane CCA1 and plane ICA4; Figure 1C). All analyses of carotid geometry were performed by one experienced rater (LT). In cases of uncertainty, another experienced rater (CS) was consulted for the final decision.

### Statistical analysis

Data are presented as mean and standard deviation or median (interquartile range) for continuous variables and as absolute and relative frequencies for categorical variables. Deviations from normality were determined using the Shapiro–Wilk statistic. Depending on data distribution, two-tailed *t*-tests or nonparametric tests were applied as appropriate for continuous variables. To detect statistically significant relations between categorical variables, a chi-square test was applied.

We used mixed-effects logistic regression models to study associations between geometric parameters and cCAPs and account for the clustering of carotid arteries per patient. For regression analysis, the geometric parameters were standardized. We used the following geometric parameters as predictors: ICA/CCA ratio, tortuosity, and bifurcation angle. Models were constructed with patient identifiers as random effects, geometric parameters as fixed-effects independent variables, and cCAP as a dependent variable. In the first step, we adjusted for age, sex, and wall area (model 1), and in the second step, we adjusted additionally for cardiovascular risk factors including hypertension, diabetes mellitus, hypercholesterolemia, and smoking (model 2). Model 3 included model 2 and all three geometric parameters. All models were adjusted for wall area because of the potential interaction of pre-existing atherosclerotic changes with lumen geometry. A two-tailed *p*-value < 0.05 was considered to indicate statistical significance. Odds ratios (ORs) and 95% confidence intervals were calculated. All analyses were performed with IBM SPSS Statistics 28.

## Results

### Study population and baseline characteristics

**Figure 2:**
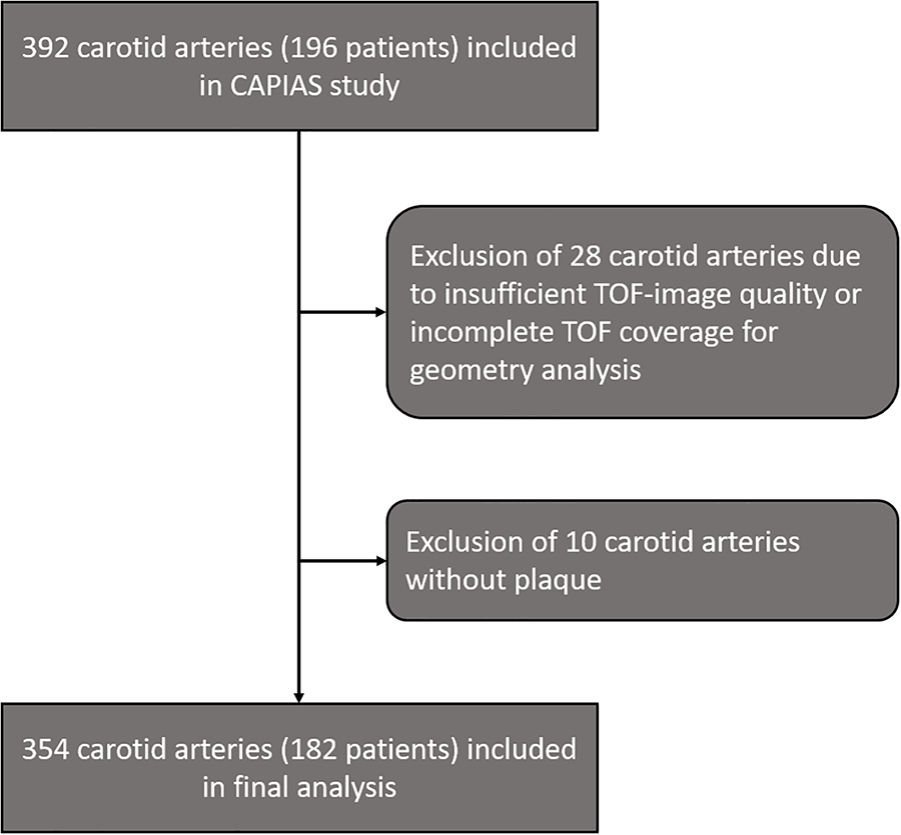
Study flowchart.

From 392 carotid arteries ipsilateral and contralateral to the ischemic infarct in 196 patients in the CAPIAS study, 354 carotid arteries (182 patients) were included in the present analysis. Carotid arteries with insufficient image quality/incomplete TOF coverage (*n* = 28) or absence of plaques (*n* = 10) were excluded (Figure 2). Baseline characteristics are provided in Table 1. Complicated plaques were found in 70 carotid arteries (19.8%). Prevalence of noncomplicated plaques was AHA-LT III in 110 (31.3%), AHA-LT IV/V in 68 (19.2%), and AHA-LT VII in 106 (29.9%) carotid arteries. Table 2 compares the geometric parameters and wall areas between carotid arteries with and without complicated plaques.

**Table 1:** Characteristics of the study cohort.

Characteristics	*N* = 182 patients
Age, years (±SD)	73.5 (±9.8)
Sex, male, *n* (%)	128 (70.3)
BMI, kg/m², (±SD)	26.5 (±3.7)
Hypertension, *n* (%)	132 (72.5)
Diabetes mellitus, *n* (%)	38 (20.9)
Hypercholesterolemia, *n* (%)	59 (32.4)
Smoking, *n* (%)	100 (54.9)
Coronary heart disease, *n* (%)	34 (18.7)
Stroke/TIA*, *n* (%)	34 (18.7)
Peripheral artery disease, *n* (%)	18 (9.9)

SD, standard deviation; BMI, body mass index; TIA, transient ischemic attack.

* Prior to the qualifying index stroke.

**Table 2:** Geometric and morphological plaque characteristics and wall areas in carotid arteries with complicated carotid artery plaques (cCAPs) defined as American Heart Association lesion type (AHA-LT) VI plaques and non-cCAPs (AHA-LT III, IV/V, VII).

Characteristics	All carotid arteries	cCAP	non-cCAP	*p*-value
	(*n* = 354)	(*n* = 70)	(*n* = 284)	
ICA/CCA ratio	0.83 (±0.18)	0.74 (±0.17)	0.85 (±0.17)	<0.001
Tortuosity	1.06 (IQR 0.06)	1.05 (IQR 0.04)	1.06 (IQR 0.06)	0.10
Bifurcation angle (°)	38.1 (IQR 29.8)	33.5 (IQR 23.3)	39.6 (IQR 30.8)	0.07
Intraplaque hemorrhage, *n* (%)	63 (17.8)	63 (90.0)	NA	NA
Juxtaluminal hemorrhage/mural thrombus, *n* (%)	51 (14.4)	51 (72.9)	NA	NA
Ruptured fibrous cap, *n* (%)	36 (10.2)	36 (51.4)	NA	NA
Wall area (mm²)	29.4 (±10.5)	36.0 (±17.2)	27.8 (±7.9)	<0.001

*P*-values represent differences between vessels with and without cCAPs.

ICA, internal carotid artery; CCA, common carotid artery; NA, not applicable.

### Association between carotid geometry and plaque composition

**Figure 3:**
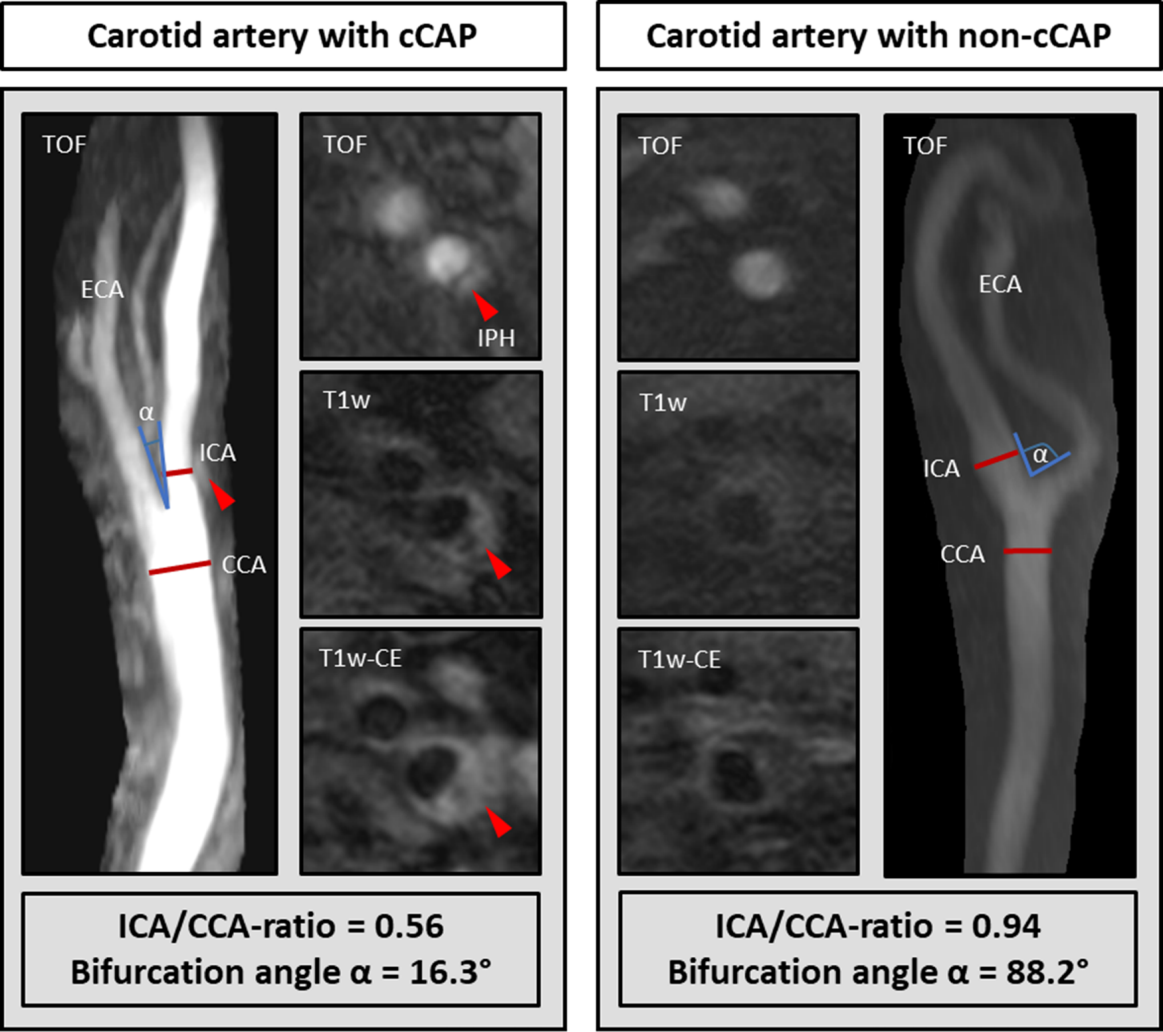
Comparison of carotid geometry in each artery with a complicated plaque (cCAP) and without a cCAP. The carotid artery with a cCAP (left) showed a lower ICA/CCA ratio and bifurcation angle *α* than without a cCAP (right). IPH, intraplaque hemorrhage.

The presence of cCAPs, i.e., AHA-LT VI plaques, was significantly associated with a low ICA/CCA ratio (OR per SD increase 0.59 [95%CI: 0.42–0.83]; *p* = 0.003) and a low bifurcation angle (OR per SD increase 0.57 [95%CI: 0.39–0.83]; *p* = 0.003) after adjusting for age, sex, and wall area (model 1). However, such an association was not existent for carotid tortuosity. These findings remained significant for the ICA/CCA ratio (OR per SD increase 0.60 [95% CI: 0.42–0.85]; *p* = 0.004) and bifurcation angle (OR per SD increase 0.61 [95%CI: 0.42–0.90]; *p* = 0.012) after additionally adjusting for cardiovascular risk factors (model 2), indicating that a carotid artery with a smaller lumen expansion of the carotid bulb (i.e., a lower ICA/CCA ratio and thus a steep vessel tapering) in relation to the CCA and a straighter course (lower bifurcation angle) is connected with a higher probability of cCAPs. By integrating all three geometric parameters (model 3), only the ICA/CCA ratio remained significant (OR per SD increase 0.65 [95%CI: 0.45–0.94]; *p* = 0.023), indicating that smaller lumen expansion may be the strongest factor associated with cCAPs. Results of mixed-effects logistic regression analysis regarding geometric parameters (i.e., ICA/CAA ratio, bifurcation angle, and carotid tortuosity) and their association with cCAPs are given in Table 3. The key findings for two carotid arteries with and without a cCAP are illustrated in Figure 3.

**Table 3:** Effects of vascular geometry on the prevalence of cCAPs.

	cCAP *n* = 70	
	OR (95%-CI)	*p*-value
ICA/CCA ratio		
Model 1	0.59 (0.42–0.83)	**0.003**
Model 2	0.60 (0.42–0.85)	**0.004**
Model 3	0.65 (0.45–0.94)	**0** **.** **023**
Tortuosity		
Model 1	0.76 (0.52–1.11)	0.155
Model 2	0.76 (0.51–1.13)	0.167
Model 3	1.03 (0.65–1.61)	0.905
Bifurcation angle		
Model 1	0.57 (0.39–0.83)	**0** **.** **003**
Model 2	0.61 (0.42–0.90)	**0** **.** **012**
Model 3	0.68 (0.43–1.09)	0.113

OR (95%CI) calculated by mixed-effects logistic regression models clustered per patient. Model 1: adjusted for age, sex, and wall area. Model 2: model 1 additionally adjusted for cardiovascular risk factors (hypertension, diabetes mellitus, hypercholesterolemia, and smoking). Model 3: model 2 and all three geometric parameters.

OR, odds ratio; CI, confidence interval; cCAPs, complicated carotid artery plaques.

## Discussion

In the present study, we investigated the association between carotid geometry and a cCAP in 354 carotid arteries of 182 patients who were part of the Carotid Plaque Imaging in Acute Stroke (CAPIAS) study. We were able to demonstrate that a smaller lumen expansion of the internal carotid artery bulb with steep tapering of the vessel into the distal ICA and a low bifurcation angle leading to a straight continuation of carotid blood flow toward the brain (i.e., low ICA/CCA ratios and low bifurcation angles) were independently associated with cCAPs after adjusting for age, sex, wall area, and cardiovascular risk factors. Thus, our findings suggest that the geometry of the carotid bifurcation independently contributes to the risk of cCAPs. Geometry shapes flow, and we believe that local hemodynamics are the underlying drivers of our observations.

### Previous investigations

Jiang et al. (17) demonstrated that a smaller luminal expansion of the carotid bifurcation, i.e., a lower “flareA” showed the strongest association with “vulnerable plaques” in Chinese patients. Vulnerable plaques in their study corresponded to AHA-LT VI and IV/V plaques with a large lipid-rich/necrotic core. In our study on Caucasian stroke patients, we used the ICA/CCA ratio, while Jiang et al. used “flareA,” the diameter ratio between distal and more proximal segments in the CCA, in a Chinese cohort. Despite these differences, we revealed similar findings; namely, that a smaller luminal expansion in the more distal segments of the carotid artery in relation to the CCA is significantly associated with complicated carotid artery plaques. Thus, our study provides further evidence that individual carotid geometry is associated with plaque composition and plaque instability. In conclusion, there is now evidence from almost 700 stroke patients from Europe and Asia indicating that carotid geometry, especially smaller lumen expansion of the internal carotid artery bulb with steep tapering of the vessel into the distal ICA, is an independent and so far under-recognized predictor for rupture-prone atheroma in the ICA.

### How can the interrelation between geometry and complicated plaques be explained?

A steep tapering of the carotid bulb into the ICA, together with a straight vessel course due to a low carotid bifurcation angle, influences the bloodstream and is likely to result in a local acceleration of blood flow velocity and pressure. Accordingly, such geometry results in an environment with high local WSS and high pulse pressure acting on the proximal shoulder of the wall and especially on the plaque surface in the case of a significant plaque protrusion. Unfortunately, we cannot provide data on hemodynamics because 4D flow MRI was not part of the protocol, but the interaction of geometry and hemodynamics has been demonstrated in several previous studies (8, 25, 26).

Carotid bifurcations with larger area ratios, i.e., larger ICA/CCA ratios, are more susceptible to low and oscillatory WSS, the so-called disturbed flow, which can be mitigated by a more curved, respectively more tortuous, vessel course (16, 26). Following flow physiology, it is plausible that a smaller lumen expansion, i.e., a steeper tapering of the vessel, reduces disturbed flow and increases WSS accordingly (17). As pointed out, the association between geometry-derived low and oscillating shear stress (8, 9, 26) is well established for the early stages of atherosclerosis. Similarly, there is growing evidence that high WSS plays an important role in the advanced stages of atherosclerosis (12, 27, 28). Combining cellular responses (including plasmin-induced metalloproteinase activity) and high WSS may lead to the degradation of matrix components, smooth muscle cell apoptosis, and reduced matrix synthesis, possibly resulting in intraplaque hemorrhage, fibrous cap thinning, and thrombus formation (10, 27, 29), as shown by Tuenter et al. (12).

As outlined before, carotid geometry also influences mechanical forces of the pulsatile blood flow, especially at the proximal shoulder of plaques and vessel stenosis. These interactions can be measured as plaque stress and tensile stress using finite-element analysis (14, 29–31). It is highly plausible that the steep tapering of the ICA in patients with complicated plaques in our cohort (i.e., low ICA/CCA ratios and low bifurcation angles) leads to high velocities that impinge on the atheroma. Accordingly, high WSS and high pressure, which are the highest in the region of maximal stenosis, act on the wall of the proximal plaque shoulder. On this side, plaque surface and volume deform with each cardiac cycle, predisposing to endothelial leakage, thinning of the fibrous cap, and intraplaque hemorrhage due to shear forces followed by vessel breakage within the plaque (15, 32, 33). This cascade leads to plaque instability and increases the risk of plaque rupture and stroke. This is supported by the observation of Jiang et al. (17) and Lu et al. (34), who demonstrated that vulnerable plaques occurred more frequently distal to the flow diverter in the ICA, i.e., at the tip or the narrowest section of the funnel.

### Limitations

We must consider some limitations of our study. First, we included carotid arteries with mild-to-moderate stenosis. More advanced atherosclerotic changes may potentially alter the geometric parameters. Thus, we corrected for wall area in our models. Even after adjusting for wall area, the association of carotid geometry and complicated plaques remained significant, suggesting that there must be other factors explaining this association beyond wall area and stenosis. In addition to the pathophysiological perspective, including mild-to-moderate stenosis is interesting from a clinical point of view, given that the prevalence of complicated plaques in symptomatic arteries is threefold higher in patients with cryptogenic stroke (5). Second, we could not interrelate geometry with WSS and plaque type to confirm our pathophysiological explanations since we did not directly measure blood flow; this would have required 4D flow MRI or CFD analysis and should have been done in future studies. Third, our geometry measurements based on 3D TOF images might be hampered due to local irregular blood flow patterns such as flow separation and recirculating flow. Finally, due to the cross-sectional study design, we can only show an association but cannot prove causality. Here, further studies with a longitudinal design are needed.

## Conclusions

In this cross-sectional, multicenter study of 354 carotid arteries from 182 patients with acute ischemic stroke, we found a significant association of carotid geometry, i.e., smaller luminal expansion of the ICA and a lower bifurcation angle, with complicated plaques. Our findings might help to identify patients at increased risk of cCAPs due to their vascular geometry, independent from other predictors such as cardiovascular risk factors and plaque composition. As this plaque type holds a high risk of future strokes , risk stratification and intensified prevention strategies for such patients based on routine CT or MR angiography or ultrasound are of high clinical interest. Furthermore, the results of our study may be valuable for future studies using artificial systems applied for blood flow simulations or engineering tools predicting blood flow hemodynamics (35). To determine the underlying mechanisms of this association, such as the interaction of high wall shear stress or mechanical stress with the plaque, further longitudinal studies using 4D flow MRI or CFD in conjunction with finite-element analysis are required.

## Data Availability

The raw data supporting the conclusions of this article will be made available by the authors without undue reservation.
